# TE-Tracker: systematic identification of transposition events through whole-genome resequencing

**DOI:** 10.1186/s12859-014-0377-z

**Published:** 2014-11-19

**Authors:** Arthur Gilly, Mathilde Etcheverry, Mohammed-Amin Madoui, Julie Guy, Leandro Quadrana, Adriana Alberti, Antoine Martin, Tony Heitkam, Stefan Engelen, Karine Labadie, Jeremie Le Pen, Patrick Wincker, Vincent Colot, Jean-Marc Aury

**Affiliations:** Commissariat a l’Energie Atomique (CEA), Institut de Genomique (IG), Genoscope, 2 rue Gaston Crémieux, BP5706, 91057 Evry, France; Centre National de Recherche Scientifique (CNRS), UMR 8030, CP5706 Evry, France; Universite d’Evry, UMR 8030, CP5706 Evry, France; Institut de Biologie de l’Ecole Normale Supérieure, F-75230, Paris, Cedex 05 France; Centre National de la Recherche Scientifique (CNRS), UMR 8197, F-75230 Paris, Cedex 05 France; Institut national de la santé et de la recherche médicale (INSERM), U1024, F-75230 Paris, Cedex 05 France; Current address: The Wellcome Trust Sanger Institute, The Wellcome Trust Genome Campus, Hinxton, Cambridge CB10 1SA UK; Current address: Technische Universität Dresden, Institute of Bota, ny, Plant Cell and Molecular Biology, D-01062 Dresden, Germany; Current address: Laboratoire de Biochimie et Physiologie Moléculaire des Plantes, Institut de Biologie Intégrative des Plantes ‘Claude Grignon’, UMR CNRS/INRA/SupAgro/UM2, Place Viala, 34060 Montpellier, Cedex France; Current address: Gurdon Institute and Department of Biochemistry, University of Cambridge, The Henry Wellcome Building of Cancer and Developmental Biology, Tennis Court Rd, Cambridge, CB2 1QN UK

**Keywords:** Transposable elements, Structural variants, *Arabidopsis thaliana*, Resequencing, DNA methylation

## Abstract

**Background:**

Transposable elements (TEs) are DNA sequences that are able to move from their location in the genome by cutting or copying themselves to another locus. As such, they are increasingly recognized as impacting all aspects of genome function. With the dramatic reduction in cost of DNA sequencing, it is now possible to resequence whole genomes in order to systematically characterize novel TE mobilization in a particular individual. However, this task is made difficult by the inherently repetitive nature of TE sequences, which in some eukaryotes compose over half of the genome sequence. Currently, only a few software tools dedicated to the detection of TE mobilization using next-generation-sequencing are described in the literature. They often target specific TEs for which annotation is available, and are only able to identify families of closely related TEs, rather than individual elements.

**Results:**

We present TE-Tracker, a general and accurate computational method for the *de-novo* detection of germ line TE mobilization from re-sequenced genomes, as well as the identification of both their source and destination sequences. We compare our method with the two classes of existing software: specialized TE-detection tools and generic structural variant (SV) detection tools. We show that TE-Tracker, while working independently of any prior annotation, bridges the gap between these two approaches in terms of detection power. Indeed, its positive predictive value (PPV) is comparable to that of dedicated TE software while its sensitivity is typical of a generic SV detection tool. TE-Tracker demonstrates the benefit of adopting an annotation-independent, *de novo* approach for the detection of TE mobilization events. We use TE-Tracker to provide a comprehensive view of transposition events induced by loss of DNA methylation in Arabidopsis. TE-Tracker is freely available at http://www.genoscope.cns.fr/TE-Tracker.

**Conclusions:**

We show that TE-Tracker accurately detects both the source and destination of novel transposition events in re-sequenced genomes. Moreover, TE-Tracker is able to detect all potential donor sequences for a given insertion, and can identify the correct one among them. Furthermore, TE-Tracker produces significantly fewer false positives than common SV detection programs, thus greatly facilitating the detection and analysis of TE mobilization events.

**Electronic supplementary material:**

The online version of this article (doi:10.1186/s12859-014-0377-z) contains supplementary material, which is available to authorized users.

## Background

TEs and their abundant relics are found in the genome of almost all organisms and are classified into many distinct families based on sequence features and transposition mechanisms [1]. DNA transposons generally exhibit cut-and-paste transposition, while retrotransposons use an RNA intermediate and thus transpose using a copy-and-paste mechanism. Retro-elements are further divided into two subclasses, depending on the presence or absence of Long Terminal Repeats (LTR). The biological role of TEs has been the subject of great controversy, and although they had been assimilated to “selfish” or “junk” DNA for some time [2], they are now recognized as important factors in the evolution of genome structure and function [3,4]. Indeed, it has been estimated that mobilization of LTR-retrotransposons is responsible for up to one tenth of spontaneous germ line mutations [5] in laboratory mice. Similarly, mobilization of the human LINE1 (L1) non-LTR retrotransposon was found to account for 19% of the structural variation between individual genomes [6], and has been linked to over a hundred human diseases [7]. In plants, bursts of TE mobilization are responsible for the large differences in genome size that are sometimes observed between closely related species [8,9].

With the advent of NGS technologies, it is now conceivable to re-sequence whole genomes in order to computationally characterize TE mobilization in a systematic way. However, this task is complicated by the inherently repetitive nature of TE sequences and by their frequent clustering in parts of the genome. Over the past years, several tools have been developed specifically for the detection of newly mobilized TEs in re-sequenced genomes [10-17]. However these tools have strong limitations. First, they all rely on prior annotation or knowledge of the TE sequence, making the detection of un- or mis-annotated TE impossible. In the same way, single transpositions involving several adjacent elements (composite events) and transposition of truncated TEs, as frequently observed in human genomes [11], are difficult to identify using such methods. Moreover, many existing tools only deal with TEs that create target site duplication (TSD) during transposition events [13,16,17], or are restricted to the analysis of the human genome (e.g. TEA [11]) or only detect the presence/absence of a TE (e.g. T-lex [12]). Finally, although several methods also attempt to identify the donor TE sequence, this identification is often limited to the subfamily level [11,15]. Therefore, exhaustive and de-novo discovery of mobilization of un- or mis-annotated TEs can only be attempted using generic SV detection tools. Four broad types of such methods have been described over the past few years. They are based on the analysis of either (i) depth of coverage, (ii) split reads, (iii) discordant paired reads, or else on (iv) de novo assembly [18]. Type (i) methods give a quantitative measure of the number of extra TE copies but do not provide information about their location. Type (ii) and (iii) methods identify one-sided events in the form of clusters of anomalously mapped reads, but they do not combine these one-sided events to produce *bona fide* TE insertions. Finally, the heavy computational burden of type (iv) methods, as well as their poor performance with repetitive sequences, preclude their use for large-scale detection of new TE insertions [19]. More recently, several programs have attempted to adopt an integrative approach by combining results from several methods [20,21], but their precision statistic is still typically low when considering specific types of structural variation (See Methods). Major drawbacks of these general-purpose tools are the fact that they produce a high number of non-TE predictions, and that none of these tools can identify the donor TE and provide the complete sequence of transposed copies.

Here, we present TE-Tracker, a new method dedicated to the systematic and robust identification of newly mobilized TEs in genomes resequenced using Illumina paired-end fragments. TE-Tracker is able to detect transposition of composite, un- or mis-annotated TEs. Moreover TE-Tracker includes a donor-scoring feature, which makes it able to detect both the identity and destination of TEs. We use TE-Tracker to provide a comprehensive view of transposition events induced by loss of DNA methylation in Arabidopsis.

## Results and discussion

### The TE-Tracker pipeline

**Figure 1:**
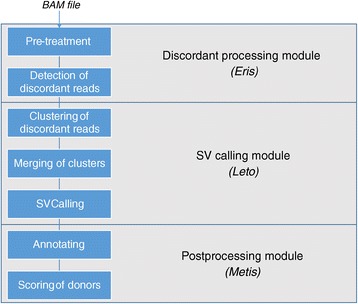
**TE-Tracker overview, main steps of the TE-Tracker pipeline.**

**Figure 2:**
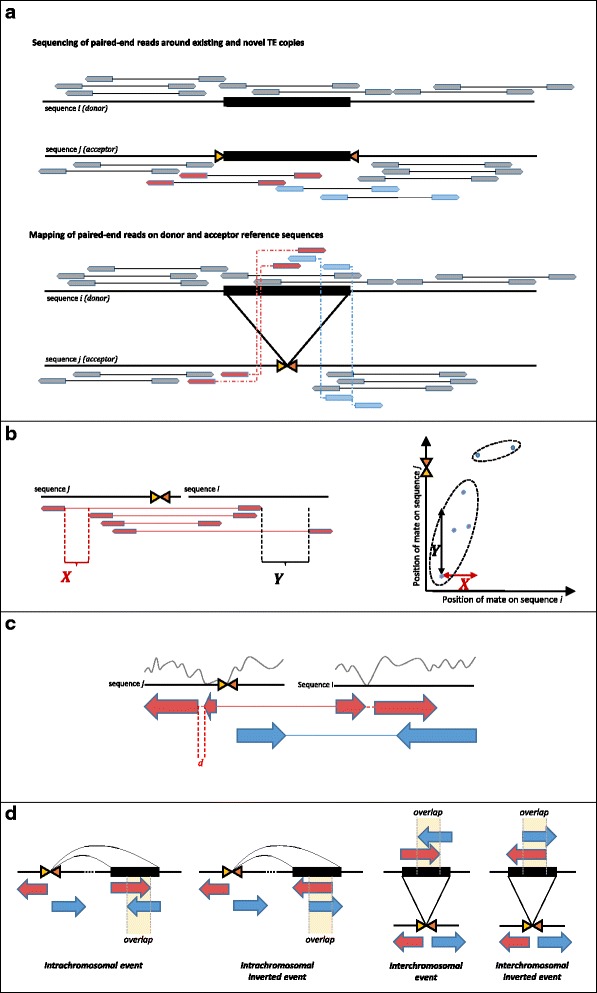
**TE-Tracker main algorithms. a**. Discordant pairs around insertion breakpoint. Sequenced reads around a newly inserted TE-copy (top half) produce discordant read mappings when aligned onto the reference sequence where the newly inserted copy only exists at the locus of origin (bottom half). The thin black line represents the sequenced DNA fragment, the thick black line represents a transposon of interest. Yellow and orange arrows represent the left and right extremities of the insertion breakpoint, linked arrows represent paired-end reads. Grey reads will be normally mapped, while colored reads will be mapped discordantly, the color indicates a type of discordance (left mate on the acceptor and right on the donor and vice-versa). **b**. Clustering of discordant pairs. Discordant reads of the same type are isolated and sorted (left half). Both ends must be sufficiently close for two read pairs to be clustered together, but sorting of the left end, combined with a random insert size results in different thresholds for clustering both ends. Pairs are clustered according to the Single-Linkage method (see Methods), which represent read pairs as edges on a graph (right half). A point is added to a cluster if its distance to any other point already in the graph meets both thresholds when projected on both axes. **c**. Cluster merging. Local drops in read coverage break clusters, corrupting insertion signals. A proximity threshold is applied to merge neighboring clusters of the same type and orientation. Local coverage is represented by a grey curve on top of the sequence, while linked colored arrows represent clusters of read pairs. **d**. Calling. The four types of transposition events detected by TE-Tracker along with their associated cluster signatures, with an emphasis on the overlap condition used to assemble clusters with compatible signatures into bona fide events.

TE-Tracker is divided into three independent modules: *Eris*, *Leto* and *Metis* (Figure 1). TE-Tracker starts with the *Eris* module detecting discordant read pairs (Figure 2a), i.e. pairs that map in unexpected orientation or location with respect to the preparation and insert size, which can constitute evidence of a transposition event. First, alignments are filtered based on mapping quality and then a random sample of the read pairs is used to estimate the insert size distribution. Median and median absolute deviation (MAD) thresholds are used to mark as discordant the pairs for which the read mates map with an unexpected insert size (see Methods). Pairs mapping on different chromosomes or in an unexpected orientation with respect to the sequencing library are flagged discordant as well. When multiple mappings are available for either mate of one pair, the pair is considered discordant only if all combinations of mate mappings match the aforementioned discordance criteria; in which case all potential mappings are recorded as if they were unique mappings from separate read pairs (see Methods).

Once discordant read pairs are extracted, they are clustered using the *Leto* module. The aim of this step is to regroup discordant pairs that might support the same transposition event while discarding lone pairs that are most certainly due to mapping errors. Clustering is done using single-linkage clustering in the mate-position space. Pairs are classified according to read orientation as well as the chromosome each mate maps on; hence for every such couple of chromosomes, each discordant pair can be represented in a two-coordinate system, making it easy to compute the respective distance between the right and left mates of any two read pairs. Clusters are built by adding pairs that are close enough to any pair already in a cluster. Because the read pairs are sorted by position, and because only the first encountered mate is ordered when sorting paired-end reads, the distance requirements for the clustering differ for both dimensions. Intuitively, the distance requirement on the ordered mate side is smaller than on the unordered mate side, since it is determined by the coverage distribution, whereas in the latter case distance is influenced by the insert size distribution, which typically has a larger variance (Figure 2b). These two values constitute the main parameters of the TE-Tracker software. In order to maximize the number of detected events, *Leto* will scan several values for both of these clustering parameters and merge clusters that are found more than once. Like discordant pairs, clusters are then classified into several types (deletion, insertion, duplication, inversion and translocation signatures*)*, according to their orientation and mapping chromosome for each mate (See Methods).

Clustering algorithms are generally memory-intensive when run over a large number of points; in particular, it is known that the optimal performance of the single-linkage algorithm used in TE-Tracker is *Ο*(*n*^2^) where *n* is the number of points [22]. In an omics context, this will result in increased computational load proportional to the number of discordant reads, either because of larger genomes or higher sequencing depth. For TE-Tracker, we choose to favor speed at the expense of memory use. For performance optimization, we developed a seed-type heuristic that reduces the amount of pairs in memory to a fraction of the total number (see Methods). Furthermore, at any given time, read mate mappings that belong to different pairs of chromosomes and are mapped in a specific orientation are considered independently and sequentially, which implies that performance of TE-Tracker will not depend on overall genome size/sequencing depth but on the average sequence size/sequencing depth for individual chromosomes. Hence, discordant reads are subdivided in up to $$ 4\times \left(\left(\begin{array}{c}\hfill k\hfill \\ {}\hfill 2\hfill \end{array}\right)\right) $$ chunks where k is the number of chromosomes. This is why performance evaluation for a pair of two chromosomes from a given species can be considered to reflect performance over that species’ genome as a whole.

Once clusters of mate pairs are formed, *Leto* attempts to merge neighboring ones (Figure 2c and see Methods) and then proceeds to call transposition events. Merging clusters is required because in regions of low coverage, the discordant read count will often be too low to allow clustering. Therefore, sudden drops in coverage can split large sets of discordant pairs into several clusters with identical signatures. Once these gaps are filled, knowledge of the dynamics of transposition and its influence on sequencing data [23] allow us to select only the combinations of cluster types that are likely to indicate a transposition event (Figure 2d). Then the program considers every combination of clusters belonging to these specific types and determines whether they could underline a true event (see Methods). For example, it takes advantage of the fact that, when the library insert size is large enough compared to the size of a mobilized sequence, the clusters anchored on the transposon side (called the donor region) will partly overlap over the middle of the TE. On the insertion site side however the corresponding ends of both clusters will be close, but will not overlap because all the reads overlapping the exact insertion site will have been left unmapped. This type of signature is a much stronger indication of a novel TE insertion than cluster proximity alone, and by applying this heuristic we manage to dramatically reduce false positive rates when calling mobilization events. The fact that TE-Tracker reconstitutes the sequence of the inserted transposon using overlapping reads allows it to fully exploit the fragment size of the sequencing library. As a result, the size of the TEs for which TE-Tracker can detect insertions and determine the donor copy is dependent on the sequencing protocol. Briefly, TE-Tracker is able to analyze mobilization of TEs that are up to 2*L* in length, where $L$ is the mean size of DNA fragments used for sequencing (see Methods). For example, in order to fully characterize the transposition landscape of Alu elements in the human genome (~300 bp), TE-Tracker would require a short fragment paired-end library of 150 bp mean length, whereas longer, recircularized fragments (such as mate-pairs) would have to be used for larger elements.

This analysis pipeline is not unlike the one used in some previous tools [24], in that it is the final heuristics step that allows incorporating constraints on clusters based on biological knowledge of insertion mechanisms. Key differences are the fact that TE-Tracker incorporates information from all mappings of a given read pair including mismatches, and that the heuristic is based on overlap of clusters alone, rather than ploidy and previous knowledge of TE donor sites.

For each pair of clusters passing the filter, TE-Tracker reports the acceptor and donor sites as defined by the cluster boundaries, the number of reads supporting the insertion event, the overlap size and whether the TE has been reversed during transposition.

**Figure 3:**
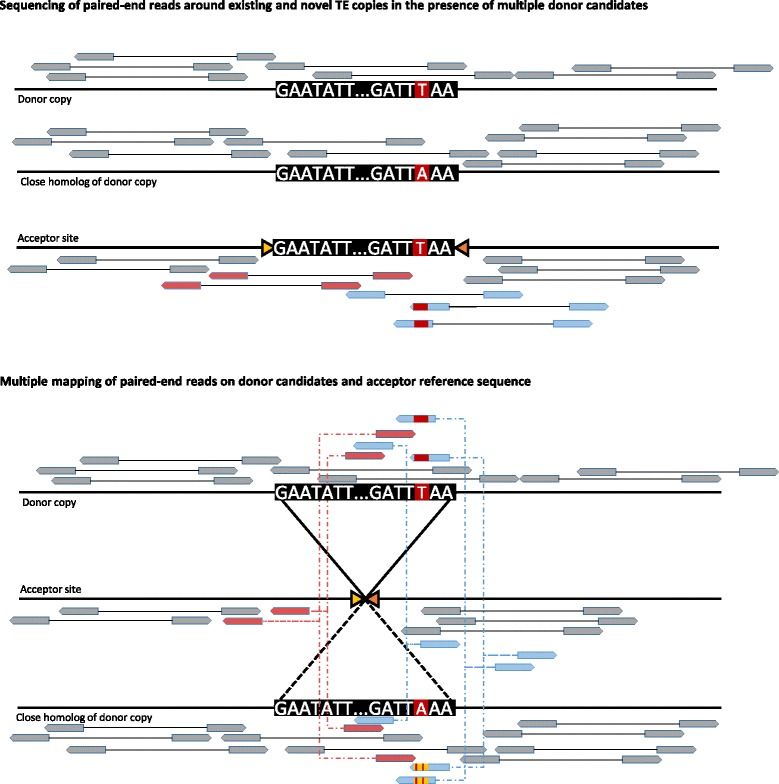
**Illustration of the donor-scoring algorithm.** In this example we describe an event involving a TE copy that differs by only one base pair from another TE in the same family. Because multiple mappings are considered, most of the discordant reads anchored around the insertion locus will map on both candidate donors equally well (plain blue and plain red reads), which will result in TE-Tracker reporting both of them. However a fraction of the discordant reads (blue reads with red mark) will span the one divergent position that differentiates both copies. These reads will map on both locations as well, but their mapping quality score will be significantly higher on the true donor copy. Counting such reads for each donor allows TE-Tracker to quickly determine a “specificity score” for each candidate, therefore helping to determinate the probable true origin of the transposition event. For simplicity, only the multiple mappings of discordant pairs were represented on this figure.

Finally, it is possible to annotate the output file with various data using the *Metis* module. If annotation data is available, both the acceptor and donor regions can be annotated; this is performed using the readily available BEDTools software suite [25]. *Metis* is also able to read a discordant BAM file such as the one produced by the *Eris* module to perform donor-scoring. Since TE-Tracker analyzes all multiple mappings of discordant pairs, it is able to report all potential donor sites for a given transposition event. However, TE families typically contain mostly defective copies that are unable to be mobilized because of truncations or other mutations in their coding or regulatory sequences. Nonetheless, potentially mobile copies are difficult to predict on the basis of sequence integrity alone, and there are no programs to date that attempt to identify those that transpose among potential candidates. Given that TE families may contain several mobile copies that differ from each other by a few sequence polymorphisms, we have included in TE-Tracker a donor-scoring feature, which selects within clusters only those reads that contain discriminating polymorphisms (Figure 3). Discordant reads anchoring at the acceptor site on one side, and at every potential donor on the other, are extracted from the input alignment file. Reads that map indifferently to all the donors are discarded, while those that map significantly better on one donor than on all the others are assigned to that donor and subsequently counted. A better mapping score on one donor location indicates coverage of a polymorphism specific to that particular TE sequence, hence the count of those specific reads for each donor represents a “specificity” or “certainty score” for that particular acceptor/donor pair. This feature aims to provide evidence in identifying the “real” donor when several candidate are available. A donor with a higher score is generally synonymous with higher specificity for that particular copy, while in cases where all of the candidate TEs have highly similar sequences, their score will be uniformly low.

### Comparison with other software

We compared TE-Tracker with RetroSeq [10], a popular program that detects novel mobilization among known TE families, as well as Delly [20], Hydra [26], VariationHunter-CommonLaw [23] and GASVPro [21], which are general-purpose structural variant detection tools that can be applied to the detection of TE insertions. We were not able to test other TE-dedicated software in a meaningful way, since only RetroSeq is generic enough to allow comparison. Indeed, it is not limited to TEs that exhibit a TSD, is not genome specific, and provides information about the family of the donor element. A comparison of the features, algorithms and input formats of all these programs is given in Table 1.

**Table 1:** **Comparison of the features, algorithms and input formats of common software used to detect mobilization of TE and/or structural variations**

**Software**	**Type**	**Input data and format**	**discordant reads detection**	**transposition calling**	**Every potential mapping considered**	**Detection of unknow TEs insertion**	**Precision**	**Donor/Acceptor output**
**TE-Tracker**		**BAM file**	**✓**	**✓**	**✓**	**✓**	**100 bp-1 Kbp**	**✓**
RetroSeq	TE-dedicated	BAM file, TE annotation or sequence	✓	✓	✗	✗	100 bp-1 Kbp	✓
Tea	TE-dedicated	BAM file, TE annotation or sequence	✗	✓	✗	✗	1 bp	✓
T-lex	TE-dedicated	FASTQ file, TE annotation	✗	✓	✗	✗	1 bp	✓
Popoolation-TE	TE-dedicated	FASTQ file, TE sequence and TSD annotation	✗	✓	✗	✗	1 bp	✓
TE-locate	TE-dedicated	FASTQ file, TE sequence	✗	✓	✗	✗	1 bp	✓
ngs_te_mapper	TE-dedicated	FASTQ file, TE annotation	✗	✓	✓	✗	1 bp	✓
RelocatTE	TE-dedicated	FASTQ file, TE sequence	✗	✓	✓	✗	1 bp	✓
TIF	TE-dedicated	FASTQ file, TE sequence and TSD annotation	✗	✓	✗	✗	1 bp	✓
VariationHunter	SV	DIVET alignment file (mrFAST output)	✗	✗	✓	✓	100 bp-1 Kbp	✓
PRISM	SV	BAM file	✓	✗	✗	✓	1 bp	✗
Delly	SV	BAM file	✓	✗	✗	✓	1 bp	✗
GASVpro	SV	Alignment file and coverage data file	✓	✗	✓	✓	100-1 kp	✗
Hydra	SV	Discordant reads coordinates and mapping features	✗	✗	✓	✓	1 bp	✗

This table illustrates a major pitfall when comparing SV detection programs, namely the variety of input file formats and level of output information. All SV detection programs will produce breakpoints, that is, clusters of reads that map anomalously on the reference genome sequence; it is the user’s task to determine which of these clusters (anchored at a given locus) can indicate a transposition event, and if it does, which of those correspond to the real donor sequence. On the other hand, RetroSeq is able to produce the insertion locus and, using prior annotation, the TE family involved; TE-Tracker will also produce a source-destination type output, but in addition it will attribute a score to potential multiple donors in an attempt to produce an unambiguous transposition signature. Moreover, TE-Tracker and the majority of other programs accept the versatile BAM alignment files, whereas VariationHunter requires a particular alignment format. Programs also differ in terms of the quantity of work they perform (Table 1): Hydra requires pre-filtering of discordant paired reads, most other programs only output breakpoints (no distinctions are made between donor and insertion sites), whereas TE-Tracker is able to do the filtering, detection and insertion calling on its own. Given this heterogeneity in the way these methods are used, we chose to harmonize the results providing equal ground for comparison (See Table 2). Finally, some programs are designed for a given sequencing protocol, e.g. short or long fragments, even if they can deal with both types of input data. In these cases, we chose to report only the results obtained from the sequencing protocol that led to the best metrics (See Table 2).

**Table 2:** **Software performance evaluated using simulated transposition events in the Arabidopsis genome**

**Software**	**RetroSeq**	**TE-Tracker**	**Delly**	**Hydra**	**GASVPro**	**Variation Hunter Common Law**
Input data^§^	PE reads	MP reads	PE reads	MP reads	PE reads	PE reads
Filter	None	>10 supporting pairs	>2 supporting pairs	>10 supporting pairs	>2 supporting pairs	>10 supporting pairs
Filtered predictions	190	351	795	10,366	6,448	26
FP	20	82	564	10,017	6,358	20
# Insertion found^†^	146	260	**282**	139	247	6
# Insertion^†^ + correct donor found	128	**244 (243)**	214	139	225	0
Positive predictive value (PPV)	67.3%	**69.5%**	26.9%	1.3%	3.5%	0%
Sensitivity	42.6%	**81.3%**	71.3%	46.3%	75%	0%

[ ^†^ ] Insertion found at +/− 300 bp.

[^§^] Paired-end (PE) reads were generated using ART and mate-pair (MP) reads were generated using SimSeqG. If programs can deal with both types of input data, we chose to report only the results obtained from the sequencing protocol that led to the best metrics.

A transposition event is qualified as « found » when at least one line in the output file has either one or the other side of a cluster overlapping the insertion site (for TE-Tracker, only the acceptor site is considered); A transposition event is qualified as « found with donor » when at least one line in the output file spans both the origin and destination sequence (for TE-Tracker the acceptor/donor nature of the site is taken into account). Even when the correct donor is identified for an insertion locus, other possible donors are often reported due to sequence similarity. For TE-Tracker, we display the number of cases where the donor-scoring feature distinguishes the real donor from all reported ones in parentheses. This feature is unique to TE-Tracker. The best detection statistic is displayed in bold in relevant rows.

In order to evaluate these programs with respect to the detection of de-novo TE insertions, we simulated 300 transposition events in the TAIR10 *Arabidopsis thaliana* reference sequence. These transposition events were classified into four subgroups : « normal » insertions correspond to events that arise from the mobilization of the full length of an annotated TE ; « composite » insertions correspond to events that mobilize a series of contiguous TEs, « long » insertions simulate the mobilization of a TE along with a certain amount of flanking sequence, and finally, « short » insertions correspond to the mobilization of a fraction of a sequence annotated as a TE (Additional file 1: Table S1). Then, we generated paired reads with a sequencing simulator. We produced the type of reads that were most suited for each program; for long-fragment paired-end reads we used the in-house SimSeqG simulator (see Methods), whereas Art [27] was chosen for short fragment paired-end reads. Simulated reads were then aligned onto the Arabidopsis reference genome sequence.

Results of the test runs are summarized in Table 2. Overall, they suggest that programs designed specifically for the detection of TE mobilization behave very differently from tools that were designed for a broader SV detection purpose. Indeed, RetroSeq, the only program in the first category, exhibits a high PPV (the number of true positives divided by the total number of events reported: 67% compared to an average 10.5% for generic tools), which translates into a significantly lower number of false positives compared to programs in the second category. However, its sensitivity is also lower, with under half (43%) of the simulated insertions successfully detected. Programs in the second category perform better in that regard (64.2% on average) but have a lower PPV. This discrepancy is a direct consequence of how each type of algorithm works: RetroSeq specifically looks for discordant pairs anchored in regions annotated as TE sequences, while the others scan the entire read space.

TE-Tracker stands between these two classes of programs, since, although it does not start from regions annotated as TE sequences, achieves a PPV (69.5%) that is slightly better than RetroSeq (67.3%). The number of true insertions found is also 78% higher with TE-Tracker compared to RetroSeq and is similar to the ones reported by Delly and GASVPro, highlighting the benefit of an annotation-independent, *de novo* approach. This is further demonstrated by the results in Table 3 in which we show the breakdown of the results according to the type of transposition event generated. As expected, RetroSeq is able to detect normal and short insertions, but performs very poorly for long and composite insertions. This suggests that RetroSeq is unable to detect mobilization events for which the TE is in fact longer than its existing annotation, or events that involve a sequence containing the annotation of two distinct TEs. TE-Tracker on the other hand exhibits similar performance over all four types of insertions, making it able to detect novel TE mobilization even in cases where pre-existing annotation is either absent, incomplete or uncertain as can be the case with complex repeated sequences such as TEs.

**Table 3:** **TE-dedicated software evaluation**

**Software**	**# Insertion** ^**†**^ **+ donor found** ^**†**^	**# Insertion** ^**†**^ **+ “normal” donor found**	**# Insertion** ^**†**^ **+ “composite” donor found**	**# Insertion** ^**†**^ **+ “long” donor found**	**# Insertion** ^**†**^ **+ “short” donor found**
RetroSeq	128 (43%)	87 (87%)	0 (0%)	0 (0%)	41 (82%)
TE-Tracker	257 (86%)	91 (91%)	81 (81%)	42 (84%)	43 (86%)

[ ^†^ ] Insertion found at +/− 300 bp.

Finally, in order to test the performance of our donor-scoring feature in the presence of a large number of potential donors, we performed similar tests on the human genome. We selected two human chromosomes, on which we simulated the mobilization of two, 6 kb-long L1-type elements that differ by 124 nucleotides and that have been described as active in the human brain [28]. In total, there are about one hundred distinct, potentially mobile full length L1 on these two chromosomes. Of the 20 random insertions generated (with random donor), 17 were detected (Additional file 2: Table S2), the three remaining ones were not detectable as they were found to have been inserted in sequence gaps. Furthermore, only one L1 donor was misattributed in this set, indicating that TE-Tracker’s donor scoring algorithm performs well even in the presence of multiple close homologs of the real donor sequence. Since TE-Tracker analyses only one pair of chromosomes at a time, the performance observed in this test can be assumed to scale to a whole-genome study.

### Application of TE-Tracker to the exploration of the transposition landscape in Arabidopsis

**Figure 4:**
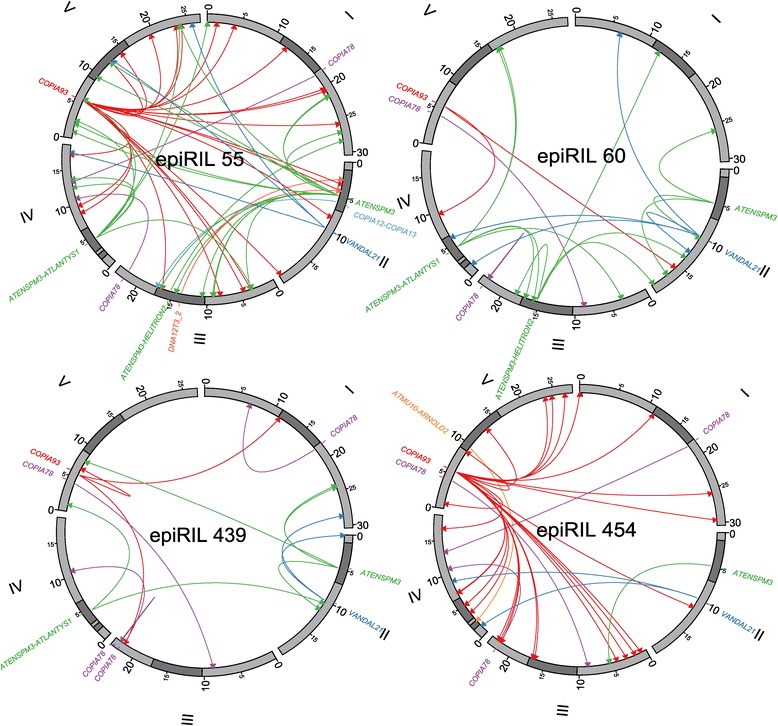
**Circos representation of new TE insertion events detected in four epiRILs.** Exterior circle represents the five chromosomes of Arabidopsis with pericentromeric regions and heterochromatic knob on chromosome 4 in dark grey. Arrows link donor TEs with the new insertion sites. Only events mapped with no ambiguity (no multiple acceptor sites and no similarity with events detected in wt) are represented.

**Figure 5:**
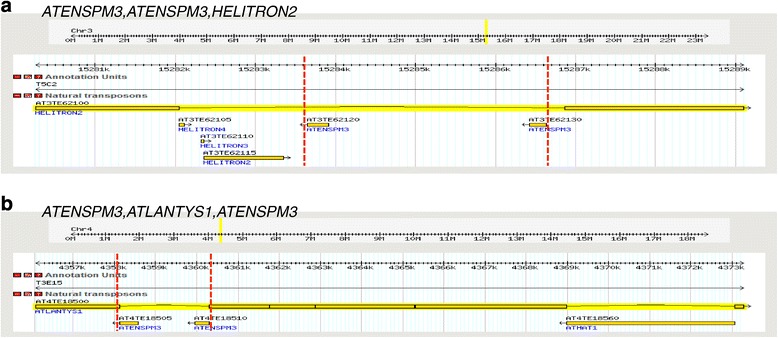
**Gbrowse view of composite elements detected by TE-Tracker.** Red dotted lines indicate the boundaries of the mobile sequence as detected by TE-Tracker. **a**. Element composed of two sequences annotated as *ATENSPM3* and one annotated as *HELITRON2*. **b**. Element composed of two sequences annotated as *ATENSPM3* and one annotated as *ATLANTYS1*.

We applied TE-Tracker to the identification of novel TE insertions in a set of four Arabidopsis epiRILs derived from a cross between a wild type (wt) plant and a mutant plant for the gene *DECREASE IN DNA METHYLATION 1* (*DDM1*) [29]. DNA methylation as well as transcriptional silencing of TEs is severely compromised in *ddm1* mutant plants [30], thus potentially leading to TE re-mobilization [31-34]. The four epiRILs together with one wt line were sequenced using Illumina mate-pair libraries (5.5 kb mean length), in order to enable the detection of new insertions for almost all of the TEs that are potentially active in the genome, as over 90% of all full-length annotated Arabidopsis TEs are less than 11 kb long [35,36]. Effective mean sequencing coverage (after alignment) ranged from 11X to 25X (Table 4). Results are illustrated in Figure 4 and summarized in Additional file 3: Table S3, Additional file 4: Table S4, Additional file 5: Table S5, Additional file 6: Table S6, Additional file 7: Table S7. Partial results obtained for several other epiRILs and using a beta version of TE-Tracker were reported elsewhere [37,38]. For the four epiRILs analyzed here, TE-Tracker could detect a total of 125 distinct insertions that match annotated TE sequences (Additional file 3: Table S3, Additional file 4: Table S4, Additional file 5: Table S5, Additional file 6: Table S6, column “Donor annotation”). The vast majority (119) of these insertions were not detected in the wt parental line, as expected if most transposition events occurred in the *ddm1* parental line or during the propagation of the epiRILs (Additional file 7: Table S7). To validate these results, a random set of 68 potentially novel insertions as well as one insertion also shared with the wt parent were tested by PCR. In all 69 cases, the presence of the insertion could be confirmed (Additional file 8: Table S8), which provides further evidence of the high specificity of TE-Tracker. Furthermore, sequencing of 26 PCR products corresponding to new insertions was used to evaluate the performance of TE-tracker in identifying donor TEs. In all but one case, the donor-scoring module was able to identify the correct TE donor sequence. Also, sequencing of both ends of 12 new insertions confirmed the presence of a target site duplication in each case, as expected for true transposition events (Additional file 9: Figure S1, Additional file 8: Table S8). Among these, we validated several insertions involving composite sequences that were not previously annotated as full-length TE units (Figure 5). These results confirm that TE-Tracker is able to detect transposition events involving sequences not explicitly annotated as a single TE, which is currently impossible with annotation-based methods such as RetroSeq [17].

**Table 4:** **Sequencing and alignment properties**

**EpiRILs**	**Number of Reads**	**−/+ (% of reads)**	**+/− (% of reads)**	**+/+ (% of reads)**	**−/− (% of reads)**	**Single (% of total)**	**Unmapped (% of total)**	**Average Mate-pair coverage (mean read depth)**	**Median fragment size (bp)**
439	127,172,830	27.6	3.6	1.5	1.6	9.0	53.7	22.3	4,900
MEJ07	92,937,978	35.2	5.3	1.4	1.4	11.8	41.9	20.8	4,900
60	85,525,387	36.0	5.2	1.4	1.4	11.2	41.5	19.5	5,200
454	92,352,477	19.0	3.1	1.1	1.1	9.0	63.8	11.2	5,300
55	71,487,300	35.9	8.0	1.8	1.8	9.4	39.9	16.3	5,300

Of the 119 distinct novel TE insertions identified, six were shared among the four epiRILs (Additional file 3: Table S3, Additional file 4: Table S4, Additional file 5: Table S5, Additional file 6: Table S6). This proportion is significantly lower than that expected (exact one-sided binomial test, p-value = 1.68e-9) if all insertions had occurred in the *ddm1* parental line used to establish the epiRIL population [29], which indicates that TE mobilization likely occurs in subsequent generations in most cases. Furthermore, transposition in the epiRILs concerns only a small number of TE families (Additional file 3: Table S3, Additional file 4: Table S4, Additional file 5: Table S5, Additional file 6: Table S6), which is consistent with a previous report of TE mobilization in *ddm1* [34]. These findings, together with the fact that most TE sequences are transcriptionally reactivated in *ddm1* [29], suggest therefore an important role of posttranscriptional mechanisms in preventing TE mobilization in Arabidopsis. Our analysis indicates in addition that mobilization, when it occurs, often concerns only one of the potentially mobile TE members of a given family. For instance, despite there being two highly similar copies of the LTR retroelement family *ATCOPIA93*, only one is detected as mobile by TE-Tracker in the genome of the Columbia accession, as was previously reported [39]. However, there are exceptions to this rule, as exemplified by the fact that several members of the LTR retroelement family *ATCOPIA78*, which is closely related to *ATCOPIA93*, have been mobilized. As many of these new *ATCOPIA78* insertions are shared among at least two of the epiRILs, transposition is likely to have taken place in most cases in the parental *ddm1* line or in the F1, which contradicts a previous claim that *ATCOPIA78* cannot transpose in this mutant background [40]. Furthermore, in the case of *ATCOPIA78* insertions, the donor-scoring feature often yielded two potential donors with similar high scores. Detailed analysis of the reads supporting one such *ATCOPIA78* insertion showed the existence of distinct sequential blocks corresponding to either donor. This is in agreement with previous reports indicating that similarly to what is seen in viruses [41], two RNA intermediates matching distinct LTR-TE family members could be encapsidated together. As a result, TE sequences could undergo recombination by template switching during cDNA synthesis [42], thus leading to the insertion of a chimeric sequence presenting block-wise similarity to both of the parent elements (Additional file 10: Figure S2). Incidentally, the validated *ATCOPIA78* insertion that is also present in the wt line may in fact reflect mis-assembly of the reference genome sequence, as this insertion maps within a truncated copy of *ATCOPIA78*. Whether the other seven TE insertions shared with the wt line also represent cases of genome sequence mis-assembly remains to be determined.

Close examination of TE-Tracker’s output revealed in addition that the DNA transposon *VANDAL21* tends to insert preferentially close to the transcription start site of genes and in the same orientation as these (9/12 instances and 8/9 instances, respectively). This result suggests that transcription initiation of the target locus is involved in the insertion of *VANDAL21* elements. Five of these *VANDAL21* insertions were tested using PCR and subsequently validated (Additional file 11: Table S10).

We also note that overall, new TE insertions are spread across the entire genome (Figure 4), which contrasts with the pericentromeric localization of most TE sequences present in the Arabidopsis genome. This suggests that purifying selection plays an important role in eliminating insertions that occur within the gene-rich regions of Arabidopsis chromosomes.

## Conclusions

We have presented a program, TE-Tracker, which accurately detects both the source and destination of novel transposition events in re-sequenced genomes. Since TE-Tracker only relies on the detection and clustering of discordant paired reads and not on TE annotation, it is generic and enables to track any mobilized TE, irrespective of its identity. Moreover, TE-Tracker is able to detect all potential donor sequences for a given insertion, and by discriminating reads that map better to a particular donor, it can attribute the correct one among them if they differ by at least one nucleotide. Furthermore, TE-Tracker produces significantly less noise than common SV detection programs, therefore allowing the researcher to focus exclusively and exhaustively on TE mobilization events in a re-sequenced genome. We have applied TE-Tracker to provide a comprehensive view of transposition events induced by loss of DNA methylation in Arabidopsis.

## Methods

### TE-Tracker algorithm

The TE-Tracker algorithm comprises three Perl modules (*Eris*, *Leto* and *Metis*) that deal with preprocessing, clustering of discordant pairs and post-processing (annotation and scoring of results), respectively (Figure 1). *Leto* is the core of the pipeline, since it wraps the *slclust* single-linkage clustering program, written in C++ using the boost library [43]. TE-Tracker’s modular architecture allows to replace each module with custom ones, provided the command-line argument set is consistent with other elements of the pipeline. Similarly, another clustering program can be used in lieu of *slclust*. TE-Tracker uses one configuration file (*SV.conf*) to manage the paths to the modules and *slclust* program, as well as all parameters used during execution. In the following sections we describe the most important steps of the pipeline, and the parameters we use on our test data.

#### Preprocessing

Each alignment file is preprocessed using the *Eris* module. Briefly, we filter the alignments using the parameter *-treat_bam = input:0–1*, which removes all mappings whose best match contain more than 1 mismatch. Depending on the quality of the sequencing and mapping, this can remove a large fraction of the reads, however we observed that this filtering did not decrease the discovery rate in our test data (Table 4), while reducing the number of false positives.

#### Discordant reads detection and classification

Let a read mapping *r(c*_*r*_*, o*_*r*_*, l*_*r*_*)* be defined by its chromosome *c*_*r*_, its orientation *o*_*r*_ (+ or -), its mapping location *l*_*r*_ on *c*_*r*_. Let a read pair mapping *p(r*_*a*_*, r*_*b*_*)*, be a doublet of two read mappings with *l*_*a*_ *< l*_*b*_. The insert size *d = l*_*b*_ *− l*_*a*_ is the distance between the two reads of *p* if *c*_*a*_ *= c*_*b*_. Let *P*_*i*_ *= p*_*i_1*_*, .., p*_*i_n*_ denote the set of *n* possible mappings for a given paired-end read pair *i*. From all the *P*_*i*_ we calculate the median *M*, the median absolute deviation *MAD,* and we define the upper (*d*_*sup*_) and lower (*d*_*inf*_) limits of *d* across all paired-end read pairs, with *d*_*inf*_ *= M −3.MAD* and *d*_*sup*_ *= M +3.MAD*.

For a large insert library, a pair mapping *p*_*i*_*(r*_*a*_*, r*_*b*_*) ∈ P*_*i*_ is mapped in a proper pair if *c*_*a*_ *= c*_*b*_ , *(o*_*a*_*, o*_*b*_*) = (−, +)* and *d*_*inf*_ *< l*_*b*_ *− l*_*a*_ *< d*_*sup*_. If such a mapping does not exist in *P*_*i*_ at least once, the pair is considered as discordant and its mapping possibilities are classified following their mapping signatures defined below. For each mapping possibilities of one read pair *pi ∈ Pi*, we have:

- *p*_*i*_*∈ Inv if c*_*a*_ *= c*_*b*_*and (o*_*a*_*, o*_*b*_*) = ((−, −) or (+, +))*
- *p*_*i*_*∈ Dup if c*_*a*_ *= c*_*b*_*and (o*_*a*_*, o*_*b*_*) = (+, −)*
- *p*_*i*_*∈ Del if c*_*a*_ *= c*_*b*_*and (o*_*a*_*, o*_*b*_*) = (−, +) and d*_*i*_ *> d*_*sup*_
- *p*_*i*_*∈ Ins if c*_*a*_ *= c*_*b*_*and (o*_*a*_*, o*_*b*_*) = (−, +) and d*_*i*_ *< d*_*inf*_
- *p*_*i*_*∈ Trans if c*_*a*_ *≠ c*_*b*_

*Del, Ins, Dup, Inv, Trans* being sets of discordant read pairs suggesting a deletion, insertion, duplication, inversion and translocation signatures, respectively [23].

Here, we consider all pair mappings as equally probable, and as such, the signal from one discordant pair is amplified by the number of its discordant mappings. This is in contrast with the probabilistic framework used in GASVPro [21], where every couple of read mappings is assigned a probability score; however our simulations (see Results) show that considering all pair mappings as equal does not increase false positive rate in the final calling.

#### Single linkage clustering and merging

We also calculated upper and lower limits of the depth of coverage *c*_*inf*_*, c*_*sup*_ using *M* and *MAD*. Pairs whose reads both map in a genomic region with a very high coverage depth (typically >1000x and containing repeated elements and low complexity sequences) are discarded from the discordant pair set. Discordant reads are sorted by *l*_*a*_ and clustered using single linkage clustering. For each subset, we built *G = (V, E)*, an undirected graph where nodes *V* are discordant read pairs. Two pairs *(p*_*i*_*, p*_*j*_*)* are linked if the distance between the two reads *r*_*ia*_ and *r*_*ja*_ is smaller than expected by coverage depth variation and if the distance between the two reads *r*_*ib*_ and *r*_*jb*_ is smaller than expected by the fragment size variation. The single linkage processus starts from a single read pair, the seed. It tries to link it to the next available pairs, when linking is not possible anymore the last linked pairs is used as seed, which is helpful in terms of computation time and memory usage. Nearby clusters with identical signatures are merged, this cluster extension allows no penalization due to low covered regions that may interrupt the linking process. After merging, the clusters are filtered by their size, rejecting those larger than d_sup_. Indeed, reads mapping around a breakpoint can only be dispersed by as much as is allowed by the insert size distribution.

#### Calling step

Intra and interchromosomal translocations are called by searching overlapping clusters of different orientation at the donor location that allows detection of translocation up to *2.M +6.MAD* bp. A deletion pattern cluster overlapping a duplication pattern cluster is needed to call an intra-chromosomal translocation in the sense donor orientation. If a deletion pattern cluster does not overlap any duplication pattern cluster and if its size is over *d*_*inf*_ , this cluster supports a deletion. An inversion pattern cluster overlapping a inversion pattern cluster of the opposite orientation is needed to call an antisense intra-chromosomal translocation. These signatures arise for both cut-and-paste and copy-and-paste transposition events, allowing TE-Tracker to indiscriminately call events involving DNA transposons or retrotransposons. For cut-and-paste events, an additional deletion cluster is expected to form around the donor copy. Since TE-Tracker also reports clusters, it is possible to manually discriminate between both types of events by looking for such clusters in the *Leto* output file.

#### Output format

*Leto* produces an unannotated, tab-separated output file, with one line per insertion event and per donor. Lines referring to different donor candidates at the same insertion site share a unique acceptor ID. Additional fields report the insertion site boundaries and the mobile element boundaries as well as the respective sizes, the cluster overlap over the donor measured in base pairs, and the number of reads supporting each particular acceptor/donor couple.

*Metis* can add up to three further columns to the output: annotation at the donor site (if available), annotation at the acceptor site (if available), and donor-scoring. The donor-scoring calculation is only calculated where applicable, i.e. in the case where multiple donors are found for a given insertion. In this case it reports the donor score for the acceptor/donor couple, else it reports a star (‘*’) character.

### Running TE-Tracker

In order to maximize sensitivity, the *Leto* module should be run over a regular grid of increasing *X* and *Y* parameters and the results pooled by traversing the grid. A step size of 50 was chosen for *X*, while a step size of 100 was chosen for *Y. X* ranged from 50 to 1000, *Y* from 100 to 5000, which amounts to a total of 1000 clustering attempts. For traversing the grid, we build a dictionary of donors from the insertions found for the couple *(X; Y)* with the largest number of insertions. Then, we go through every point of the grid and add data to the dictionary: we perform the cartesian product of the dictionary and the output file and add events that do not overlap either on donor or acceptor site. This allows to build a comprehensive landscape of all insertions that appear at least once on the grid. When an event is found in several points of the grid, the one supported by the most reads is kept, which ensures that the optimal clustering parameters for each insertion were used for each line of the final output file.

### Comparison with other software

#### Synthetic genome simulation

We performed tests on simulated data to assess theoretical sensitivity and specificity. We simulated 300 transposition events (See Additional file 1: Table S1) in the TAIR10 *Arabidopsis thaliana* reference sequence. Four types of events were generated:

- “normal” insertions correspond to events that arise from the mobilization of the full length of a TE ;
- “composite” insertions correspond to events that mobilize a series of contiguous TEs ;
- “long” insertions simulate the mobilization of a TE along with a certain amount of flanking sequence ;
- “short” insertions correspond to the mobilization of a fraction of a sequence annotated as a TE.

#### Short fragment paired-end reads simulation

Art [27] was chosen for simulate short fragment paired-end reads from the tampered reference sequence and the TAIR10 reference sequence.

#### Long fragment paired-end reads simulation

We used the in-house SimSeqG software to simulate long fragment paired-end reads from the tampered reference sequence and the TAIR10 reference sequence. The SimSeqG simulator aims to reproduce the position-dependent sequencing error rate, short-fragment paired-end contamination and chimeric read rate found in a particular long-fragment paired-end sequencing. As such, a first phase will draw a sample from a BAM file and compute several descriptive statistics, this will allow to calculate two of the three error rates and apply it to the second phase, which is the simulation in itself. The first phase proceeds as follows:

- For each base position, the software will calculate the empirical probabilities of observing all possible quality values. This results in the computing of as many histograms as there are bases in a read, the number of classes in each histogram being equal to the number of possible base qualities according to the standard used (phred-33 or phred-64)
- The software will compute the insert size distribution of all unambiguously mapped pairs, which usually yields a bimodal distribution corresponding to a mixture of 1) the short fragment contamination and 2) the long-fragment library of interest. It will then use the minimum separating the two modes of the distribution and the ratio of the modes themselves to infer the odds of obtaining a short or long fragment for a random read sample.

The simulation phase is designed to mirror the sequencing process as closely as possible:

- A fragment size is sampled from the empirical distribution;
- a location is randomly selected in the genome and a sequence corresponding to the sampled fragment length is extracted starting at this position;
- the fragment is circularized and a random splice length is chosen from the short fragment length distribution;
- a splice start is randomly chosen around the circularization point and the sequence is extracted from the circularized fragment;
- since the splice start is random, it will sometimes fall close enough to the circularization point for the read length to extend over it, which will generate a chimeric read;
- both ends of the subfragment are extracted and sequenced: for each base, the program will draw a quality corresponding to its position from the empirical quality distribution. Then, it will produce a sequencing error at that position with probability given by the base quality.
- once in a while, at a rate determined from the BAM learning set, a configuration leading to the production of a parasitic short fragment is produced and the result is sequenced in a similar way;
- reads and quality values are then written in FASTQ format.

#### Read mapping

Simulated reads were aligned unto the TAIR10 *Arabidopsis thaliana* genome with BWA v.0.6.1 [44], using the parameters -R 10000 -l 35 -O 11 in aln, and –ns–N 10000 in sampe for short fragment paired-end reads and parameters -R 10000 -l 35 -O 11 in aln, and –n 10000 –N 10000 in sampe for long fragment paired-end reads.

#### Benchmarking of the donor-scoring feature

Two L1 elements located on human chromosome 12 (chr12:75268648–75274681 and chr12: 101539821–101545842) were selected for this test. For each of these two donors, we simulated 10 random, full-length insertions on the b37 reference sequence of human chromosome 19. SimSeqG was used to simulate long-fragment mate-pair reads on the modified reference sequence of chr19 and on the untampered reference sequence of chr12. Error rates and chimeric read rates used by SimSeqG were learned from the alignments of the four Arabidopsis reads sets. Then, reads were aligned using the same parameters as before and TE-Tracker was run on the alignments. For performance reasons, only one run was considered instead of scanning the whole clustering parameter space.

### Genomic DNA sequencing and mapping

DNA was extracted from seedlings grown under long-day conditions, using DNeasy Qiagen kits. About 10 microgram of genomic DNA were sonicated separately to a 4–6 Kb size range using the E210 covaris instrument (Covaris, Inc., USA). Libraries were prepared following Illumina’s protocol (Illumina Mate Pair library kit). Briefly, fragments were end-repaired and biotin labeled. A size selection of fragments with length of interest (around 5 Kb) was performed. DNA were then circularized and linear, non-circularized DNA were eliminated by digestion. Circularized DNA were fragmented to 300-700-bp size range using covaris E210. Biotinylated DNA were purified, end-repaired, then 3′-adenylated, and Illumina adapters were added. DNA fragments were PCR-amplified using Illumina adapter-specific primers. Finally, the PCR amplified libraries (350–650 bp) were size-selected. Libraries were then quantified using a Qubit Fluorometer (Life technologies) and libraries profiles were evaluated using an Agilent 2100 bioanalyzer (Agilent Technologies, USA). Each library was sequenced using 100 base-length read chemistry in a paired-end flow cell on the Illumina GAIIx (2 lanes) or HiSeq2000 (1 lane) (Illumina, USA).

### Read mapping

Reads were then mapped with BWA v.0.6.1 [43], using the parameters -R 10000 -l 35 -O 11 for aln, and the parameters –n 10000 –N 10000 -s for sampe, onto the TAIR10 reference sequence [45]. Reads hanging over chromosome ends were removed using picard CleanSam, duplicate pairs were removed using picard MarkDuplicates [46].

### Filtering out ambiguous acceptor sites from TE-Tracker outputs

Read mapping to the reference Arabidopsis genome sequence revealed several regions with extremely high coverage, which correspond mainly to the centromeric repeat unit of 180 bp and the rDNA 45S and 5S repeat units. These as well as the few regions with low sequence complexity were removed from the TE-Tracker output as insertions could not be mapped with any confidence in these regions. Briefly, read-depth (RD) was calculated on consecutive non-overlapping windows of 100 bp. After correction for GC content bias [47], consecutive windows (allowing one window gap) with a RD more than three MAD from the median RD signal were merged to define larger segments and acceptor sites overlapping with segments longer than 500 bp were excluded from the TE-Tracker output (Additional file 12: Table S9 and Additional file 11: Table S10; a total of 1,125,487 bp or 0.94% of the reference Arabidopsis genome sequence).

### PCR validation

A list of primers used for the validation of detected insertions is provided in Additional file 13: Table S11.

### Availability of supporting data

The data sets supporting the results of this article are available in the European Nucleotide Archive (ENA) repository under accession: ERS389787 (epiRIL 60), ERS389793 (epiRIL 55), ERS392388 (epiRIL 454), ERS392386 (epiRIL MEJ07) and ERS392380 (epiRIL 439).

## Additional files

Additional file 1: Table S1. Insertions generated for simulated sequencing. 300 artificial TE insertions were generated with a random acceptor site. Type “T” indicates that the mobilized donor sequence represents the full length of an annotated TE unit, “C” indicates that two consecutive TE annotation units were mobilized, “S” indicates that only a part of the annotated TE unit was mobilized and “L” indicates the mobilization of a sequence that comprises a TE unit, but extends beyond it. Additional file 2: Table S2. Simulated insertions between two human chromosomes. Additional file 3: Table S3. Insertions detected by TE-Tracker in epiRIL 55. Additional file 4: Table S4. Insertions detected by TE-Tracker in epiRIL 60. Additional file 5: Table S5. Insertions detected by TE-Tracker in epiRIL 439. Additional file 6: Table S6. Insertions detected by TE-Tracker in epiRIL 454. Additional file 7: Table S7. Insertions detected by TE-Tracker in wt. Additional file 8: Table S8. Summary of PCR validation of new insertions. Additional file 9: Figure S1. PCR primer design for the validation of insertions detected using TE-Tracker. Additional file 10: Figure S2. Example of transposition event detected by TE-Tracker that might involve a chimeric element containing sequences from 2 distinct donors. Additional file 11: Table S10. Raw TE-Tracker output for the four *Arabidopsis* lines. Additional file 12: Table S9. Regions masked for TE detection. Additional file 13: Table S11. Primer sequences for PCR validation of new TE insertions detected with TE-Tracker.

## Abbreviations

SV: Structural variation
EpiRIL: Epigenetic recombinant inbred line
MAD: Median absolute deviation
TE: Transposable element
SNP: Single nucleotide polymorphism
NGS: Next generation sequencing technologies
DDM1: Decrease in DNA methylation 1
SLC: Single linkage clustering
RD: Read depth
