# Supplementary material for: TE-Tracker: systematic identification of transposition events through whole-genome resequencing
Source: BMC Bioinformatics. 2014 Nov 19;15(1):377. doi: 10.1186/s12859-014-0377-z (PMC4279814; doi:10.1186/s12859-014-0377-z)
Supplement: Additional file 9: Figure S1. — PCR primer design for the validation of insertions detected using TE-Tracker. [file 12859_2014_377_MOESM9_ESM.pdf]

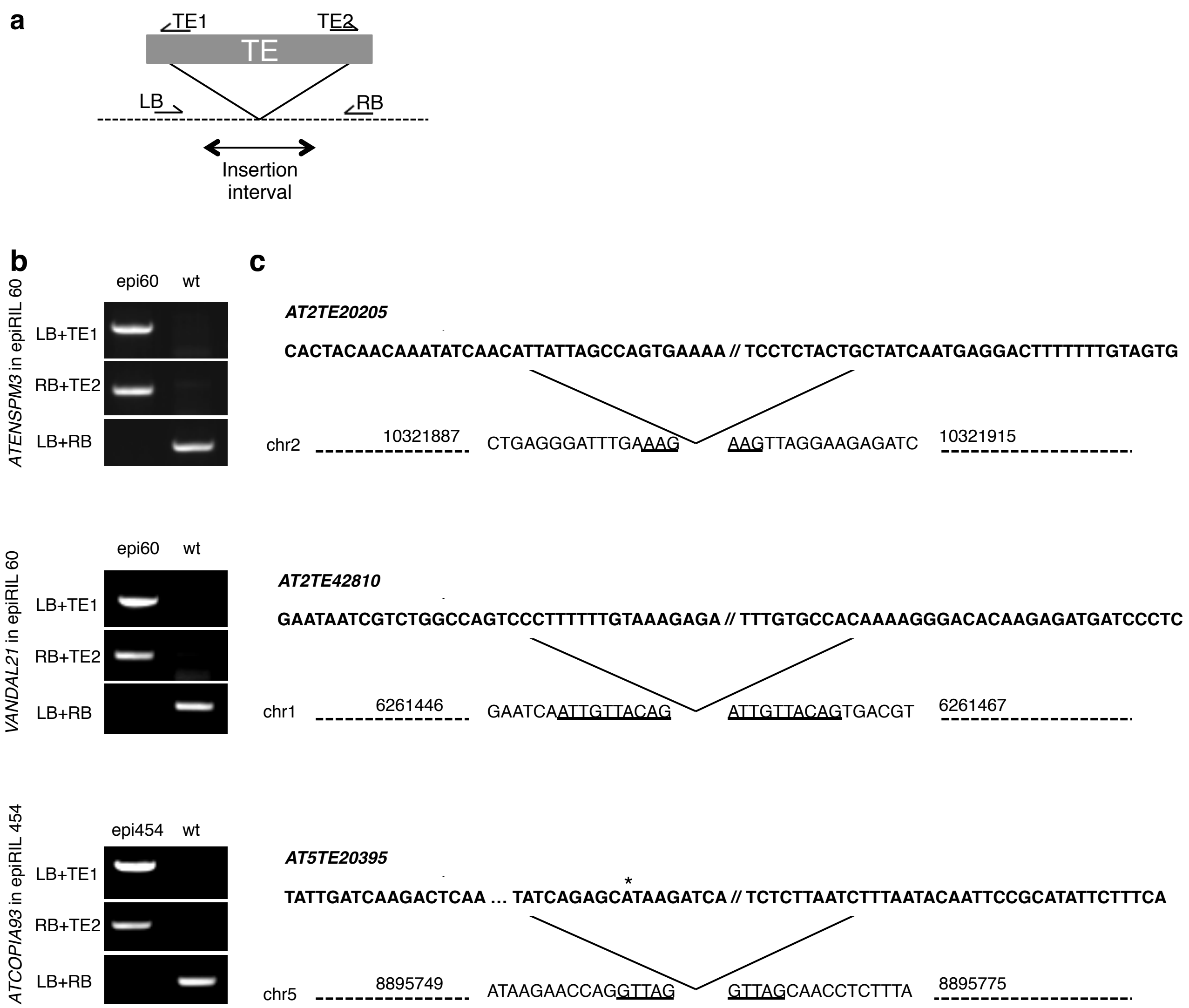

Supplementary Figure 1

Validation of new TE insertions. a. Scheme of the PCR validation procedure, arrows represent primers. **b.** PCR results for 16 three new insertions identied in two epiRILs and involving three distinct donors : ATENSPM3 (AT2TE20205), VANDAL21 (AT2TE42810) and ATCOPIA93 (AT5TE20395). **c.** Sequencing results of the PCR products that correspond to the borders of new insertions. TE sequence is represented in bold and target site duplications generated by TE insertion are underlined. The star highlights the single polymorphism located in the extremity of the TE and that discriminates between the two ATCOPIA93 potential donors.
