# Supplementary material for: TE-Tracker: systematic identification of transposition events through whole-genome resequencing
Source: BMC Bioinformatics. 2014 Nov 19;15(1):377. doi: 10.1186/s12859-014-0377-z (PMC4279814; doi:10.1186/s12859-014-0377-z)
Supplement: Additional file 10: Figure S2. — Example of transposition event detected by TE-Tracker that might involve a chimeric element containing sequences from 2 distinct donors. [file 12859_2014_377_MOESM10_ESM.pdf]

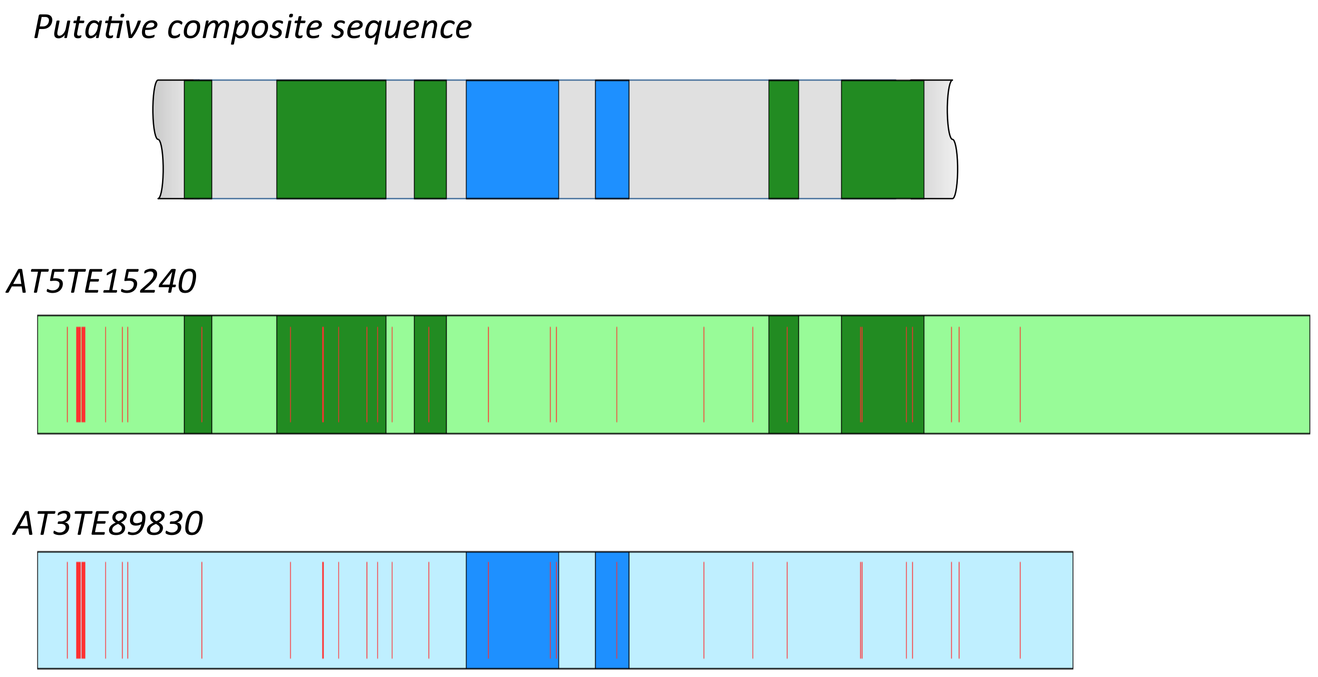

Supplementary Figure 2

Multiple-origin unique regions for donor suggest a recombination-like event during transposition. Clusters of reads mapping to acceptor region chr3:8763824..8765272 of epiRIL 454 have their mates mapping on several genomic regions corresponding to ATCOPIA78 sequences, which indicates multiple potential donors. The donor scoring feature extracted mates that mapped significantly better on one copy than on all others; this yielded not one, but two candidates. Piling up the said reads led to the configuration above. In light blue and green are the two copies, aligned unto each other. Red ticks indicate variants (SNP and indels) between the two copies. In dark blue and green are the regions delimited by specific reads for each copy, respectively. On top is the putative inserted sequence with blocks coming from either copy. Ticks not included in darker regions represent non-unique variations that are therefore also found when aligning to other COPIA78 sequences.
